# Development of a Computational Model of Abscess Formation

**DOI:** 10.3389/fmicb.2018.01355

**Published:** 2018-06-26

**Authors:** Alexandre B. Pigozzo, Dominique Missiakas, Sergio Alonso, Rodrigo W. dos Santos, Marcelo Lobosco

**Affiliations:** ^1^Department of Computer Science, Federal University of São João Del-Rei, São João Del-Rei, Brazil; ^2^Department of Microbiology, University of Chicago, Chicago, IL, United States; ^3^Department of Physics, Universitat Politècnica de Catalunya, Barcelona, Spain; ^4^Graduate Program in Computational Modeling, Federal University of Juiz de Fora, Juiz de Fora, Brazil

**Keywords:** *S. aureus* infection, abscess formation, fibrin network, partial differential equation, computational modeling

## Abstract

In some bacterial infections, the immune system cannot eliminate the invading pathogen. In these cases, the invading pathogen is successful in establishing a favorable environment to survive and persist in the host organism. For example, *S. aureus* bacteria survive in organ tissues employing a set of mechanisms that work in a coordinated and highly regulated way allowing: (1) efficient impairment of the immune response; and (2) protection from the immune cells and molecules. *S. aureus* secretes several proteins including coagulases and toxins that drive abscess formation and persistence. Unless staphylococcal abscesses are surgically drained and treated with antibiotics, disseminated infection and septicemia produce a lethal outcome. Within this context, this paper develops a simple mathematical model of abscess formation incorporating characteristics that we judge important for an abscess to be formed. Our aim is to build a mathematical model that reproduces some characteristics and behaviors that are observed in the process of abscess formation.

## 1. Introduction

In some *Staphylococcus aureus* infections, neutrophils cannot completely eliminate the invading pathogen. In such cases, a lesion known as abscess may form, especially in skin or in soft tissue organs. An abscess is characterized by an area comprising invading pathogens, fibrin, immune cells (mainly neutrophils) and many types of dead cells, and it may be formed in response to viral or bacterial infections in various organs. Abscess formation is often a defense mechanism elicited by the host to prevent dissemination of pathogens. However, in some instances, such as mycobacterial and staphylococcal infections, the pathogen appears to have subverted this defense and paradoxically uses this environment to thrive and persist (Cheng et al., 2009, 2010; Graves et al., 2010; Kim et al., 2011, 2012; McAdow et al., 2012).

Following intravenous infection of mice, *S. aureus* starts to leave the vasculature to colonize the renal tissue a few hours later. In the vasculature, *S. aureus* begins to produce toxins^1^. Some, like α-toxin, can target various cell types and lead to massive damage in infected sites. Other, like the leukotoxins, are more specific and target mainly leukocytes (Kwiecinski, 2013). The function of these toxins is thought to primarily kill immune cells, but also to alter host responses. For example, interaction of α-toxin with its receptor ADAMS10 causes tissue barrier disruption that may facilitate dissemination from the vasculature to organs (Berube and Bubeck Wardenburg, 2013). *S. aureus* also induces the clotting of blood and plasma in the vasculature (Cheng et al., 2009, 2010). Presumably this mechanism prevents immune cells, in the bloodstream, to phagocytose the bacteria. Further, this mechanism is responsible for the formation of bacterial agglutinates or micro-emboli that may help to mechanically disrupt the endothelial barrier and thereby allow the bacteria to gain access into tissues. Despite these strategies, few bacteria manage to survive in the vasculature and establish lesions in the kidney successfully. Within 3 h of infection, the bacteria load in both blood and kidneys are high (Cheng et al., 2009, 2010). Then bacteria loads decrease until 12 h post inoculation (Cheng et al., 2009, 2010). This is due to the fact that immune cells, mainly neutrophils, are successfully eliminating the majority of bacteria. Other host defense mechanisms, such as complement system, also contribute to bacterial killing (Foster, 2005). Then after 12 h, we can clearly view a pattern of logistic growth of the bacteria load. This pattern appears as a result of the abscess formation dynamics (Cheng et al., 2009).

After 12 h, *S. aureus* starts to replicate forming a *Staphylococcus* abscess community (SAC) inside the abscess lesion. During this process, the bacteria employ a variety of mechanisms to kill and evade immune cells. But equally important is a mechanism used by *S. aureus* to isolate themselves from immune cells conferring an even greater protection. This mechanism is the result of the deposition of fibrin clots around the SAC, and around the entire lesion (Cheng et al., 2009, 2010; McAdow et al., 2012). *S. aureus* secretes coagulases, Coa and vWbp, that bind to and activate prothrombin, thereby converting fibrinogen to fibrin. The coagulases diffuse throughout the tissue from the SAC, inducing the conversion of fibrinogen to fibrin in the regions around the bacteria colonies. As a result, a fibrin network is formed around the SAC (Foster, 2005; Cheng et al., 2010; McAdow et al., 2012). *S. aureus* encodes a surface protein called Clumping Factor A (ClfA) (Foster and Höök, 1998), which is responsible for the recognition and binding to fibrin. ClfA-mediated binding of fibrin delineates the first margin of the SAC. The resultant fibrin polymer forms the structure of fibrin around the staphylococci (Foster, 2005; Cheng et al., 2010; McAdow et al., 2012), and *S. aureus* persists in the center of abscess lesions protected from the immune system. Unless staphylococcal abscesses are surgically drained and treated with antibiotics, disseminated infection and septicemia produce a lethal outcome (Kim et al., 2011). Therefore it is important to gain a deep understanding of how an abscess is formed in order to develop vaccines and treatments to *S. aureus* infections. *In vivo* experiments have been performed to identify the factors necessary for abscess formation, but the search for its determinants is a complex task, since it requires studying the interaction between hundreds or even thousands of components that participate in the process and analyzing how observed behavior emerges from these interactions. Mathematical and computational modeling (Bender, 2000; Meerschaert, 2013; Shiflet and Shiflet, 2014) can help in this search, contributing to a better comprehension of some aspects of abscess formation as, for example, the importance of different mechanisms employed by pathogens to survive in the host.

A set of related works developed mathematical models of the immune response with the objective of studying the following subjects: (1) the innate immune response to a bacterial infection, (2) the formation of bacteria colonies, and (3) the dynamics of interaction between the host and the pathogen. The related works bear some similarities to this paper, such as for instance, the modeling of bacteria and neutrophil cells and the modeling of processes such as bacteria replication, neutrophil migration, phagocytosis and diffusion. However, none of them are capable of reproducing the formation of a stable abscess pattern.

In Keener and Sneyd (1998) a unidimensional model developed by Alt and Lauffenburger (1987) is presented to study under what conditions Polymorphonuclear leukocytes (PMNs), more commonly called neutrophils, are successful in controlling a bacterial infection. The model is comprised of three variables: bacteria (b), cytokine (c) and neutrophil (n). The authors performed a linear stability analysis of the model [more details can be obtained in section 16.3 of the book Mathematical Physiology Keener and Sneyd, 1998] and the results obtained can be summarized in three cases: (1) bacteria are completely eliminated and the neutrophil concentration stabilizes to a normal value; (2) neutrophils cannot control the growth of bacteria and bacteria grow without limitation; (3) neutrophils control the growth of bacteria, but they cannot completely eliminate them. In this case, there is a state of persistent infection where both are present and maintain a balance. These three behaviors are also obtained in the bacteria-neutrophil model developed here. The paper concludes that a bacterial infection can be controlled when the rate of phagocytosis is sufficiently large and the immune response is most effective when neutrophils are able to recruit more cells and move chemotactically. As will be shown, the same behavior is observed in this paper for models that consider the dynamics of neutrophils. The model of Alt and Lauffenburger (1987) does not consider the dynamics of fibrin as this paper does. Here, we study and analyze the effects of fibrin in a mathematical model of the abscess formation process.

Kawasaki et al. (1997) have developed a reaction-diffusion system for bacterial and nutrient concentrations that reproduces various observed growth patterns in colonies of bacteria. One of the important elements of the model is a non-linear diffusion term that depends on both concentrations of bacteria and nutrients. The model simulates the fact that, in regions devoid of nutrients, the bacteria cannot move, becoming more inactive. They were able to produce highly branched patterns only with the presence of a minimal anisotropy coming from the square *lattice* used in simulations. In spite of reproducing several patterns, the model was not able to reproduce the pattern of concentric rings because, according to the authors, this pattern requires additional mechanisms. The model of Kawasaki et al. (1997) does not study the immune response to a bacterial infection, the dynamics of fibrin and toxins as this paper does. Besides, the model does not consider diffusion to be dependent on the amount of available space as the models presented in this paper do.

An additional mechanism was proposed by Lacasta et al. (1999). They presented a model of reaction-diffusion for the growth of colonies of bacteria of the species *Bacillus subtilis*. The model is comprised of two equations for the concentrations of bacteria and nutrients. Like the previous model of Kawasaki et al. (1997), the model of Lacasta and co-authors was able to reproduce different growth patterns of species *B. subtilis*, which resulted in a rich variety of structures. Certain structures, such as concentric rings, were only obtained because they considered in the model a cooperative behavior among bacteria. This behavior was modeled considering a global phenomenological variable that represents the number of bacteria most active in the colony, that is, the bacteria that move more in search of nutrients. In addition, they considered a nonlinear diffusion coefficient that depends on this variable. Lacasta et al. (1999) did not consider the immune response, the dynamics of fibrin and toxins in their model as this paper does.

Smith et al. (2011) developed a number of models to gain a greater understanding of how different layers of host defense in the lower respiratory tract, including resident cells and recruited cells, combine to form a response against a pneumococcal lung infection. In this study, the immune response is divided into three stages: (1) the response given by resident alveolar macrophages; (2) the response given by neutrophils; and (3) the response given by macrophages derived from monocytes from the bloodstream. Mathematical models that describe the dynamics of each of these three stages were developed (Smith et al., 2011). Smith and co-authors studied the relationship between the inoculated concentration of bacteria and two outcomes: (1) the establishment or (2) the eradication of an infection. First, they used a single alveolar macrophage response equation to study how a threshold dose determines whether the result will be the establishment or eradication of the infection. This model was then extended to incorporate pro-inflammatory cytokine production accompanied by neutrophil recruitment. Finally, they examined the possibility of elimination of the bacteria given by an influx of monocyte-derived macrophages. The authors argue that through these models it was possible to better understand the contribution of each of the variables considered for the initiation and resolution of pneumococcal pulmonary infection and were able to capture the qualitative behavior of the experimental data. The work of Smith et al. (2011) does not consider the dynamics of fibrin formation and toxin production by the bacteria and the interactions between fibrin, toxin and neutrophils.

Other studies examine the dynamics of parasites in the immune system. The first work (Antia et al., 1994) considers the dynamics of parasites during an acute infection. The model considers a generic population of parasites and it assumes that the virulence of parasites is proportional to the rate of parasite growth in the host. The results indicated that the transmission would be more efficient if the parasite had an intermediate growth rate (not as high as, for example, *E. coli*, and not as low as *M. tuberculosis*). The authors argued that this would result in an evolution and maintenance of an intermediate level of parasitic virulence. A second work by Antia et al. (1996) considered a different set of hypotheses for the dynamics of persistent parasitic infections. This model predicts that initial persistence in the host can be achieved by parasites that grow very slowly or by parasites that have a niche that is inaccessible to the immune response. In addition, the authors suggested that the evasion of immune response by the pathogen at a time well after the onset of infection may be a consequence of two processes: (1) deletion of T cells in the thymus caused by the antigens; and (2) presence of a maximum limit on the number of divisions of a T cell. In this paper, we show that a refuge mechanism used by some bacteria to persist in the host is the formation of a fibrin network that confers protection against the immune response.

In our previous paper (Pigozzo et al., 2012), we were capable of reproducing the initial formation of an abscess, but the abscess pattern did not remain stable. One possible explanation is the fact that *S. aureus* abscesses are encapsulated within a fibrin capsule triggered upon secretion of two coagulases, Coa and vWbp (Cheng et al., 2010; McAdow et al., 2012), which were not modeled in our previous paper.

**Figure 1:**
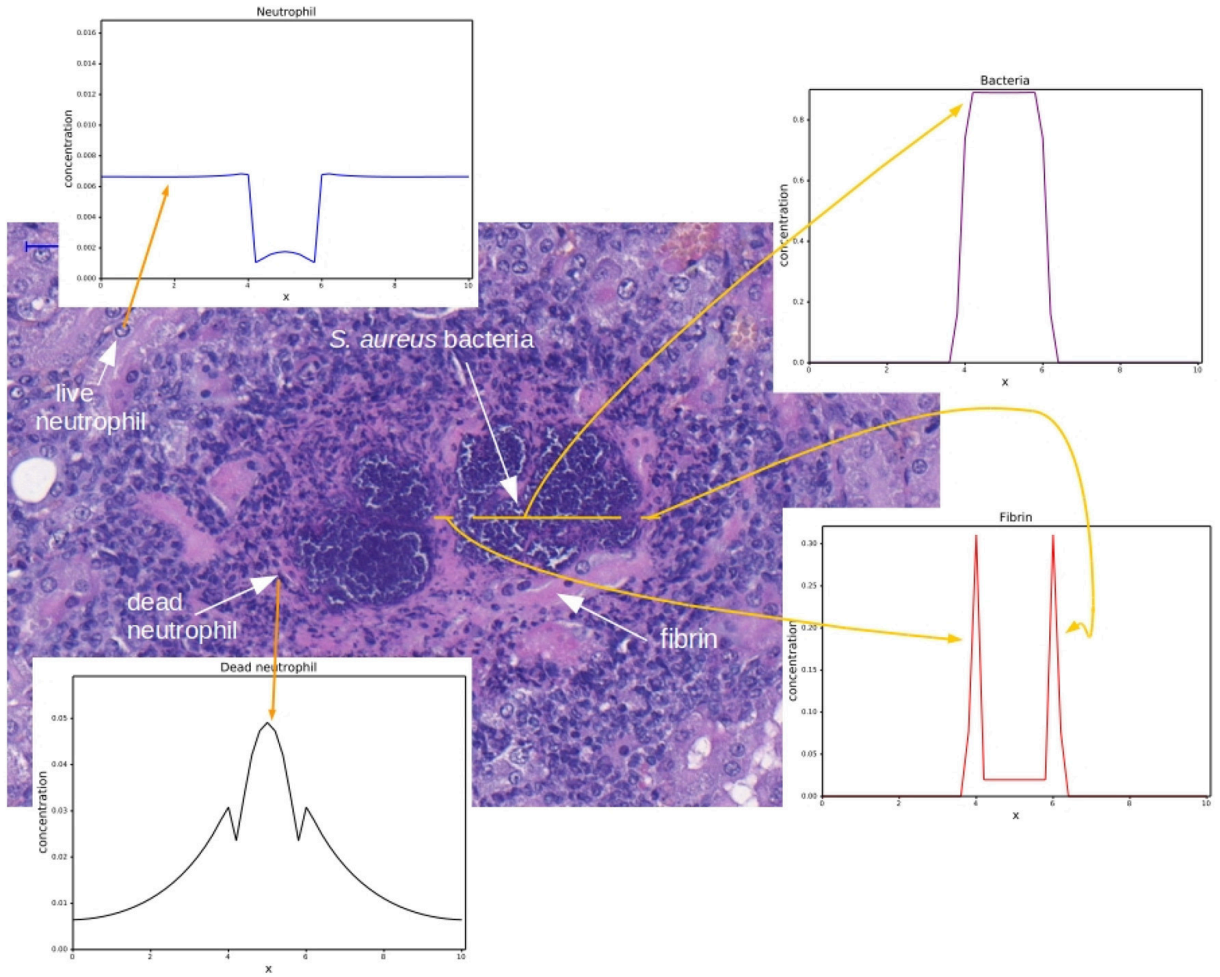
Histology image provided by the Laboratory of Microbiology of the University of Chicago. Adapted with permission of Dominique Missiakas and Olaf Schneewind. The histology image shows a mouse renal tissue infected with *S. aureus* and the corresponding spatial distributions for each cell type. The spatial distributions were obtained by the computational simulation that will be presented in this paper. The darker purple region is the colony of staphylococci and the pink region around the staphylococci colony is the fibrin network. Some dark points around the fibrin are necrotic neutrophils and some points in the “periphery” of the abscess are live neutrophils. These regions form the abscess. For each region highlighted in this figure, an example of a result obtained by the implementation of the mathematical models presented in this paper is shown with a yellow arrow.

The objective of this paper is to construct a mathematical model, based on partial differential equations (PDEs), that essentially reproduces a pattern that is observed in histology images of renal abscesses in mice (Cheng et al., 2009, 2010; Graves et al., 2010; Kim et al., 2011; McAdow et al., 2012; Kim et al., 2012). The pattern is comprised by the following regions: )1) some region occupied by the bacteria colony (SAC); (2) some region containing fibrin that forms a network around a bacteria colony; and (3) surrounding the fibrin network, a region comprised mainly of necrotic neutrophils and some live neutrophils. Figure 1 shows these regions and how they appear in the results of the computational simulations of this paper. In addition, we study and analyze the characteristics of distinct models involving the interactions between bacteria, the two coagulases or coagulation factors, Coa and vWbp, fibrin and neutrophils. This paper shows that it is possible to reproduce some aspects of abscess formation through computational models that are able to capture the spatiotemporal dynamics of the fibrin network formation around the bacteria colony as well as the neutrophil response to the bacterial infection. The computational models were implemented using an explicit Euler method for time discretization and, for the spatial discretization, the Finite Volume Method (Versteeg and Malalasekera, 2011), as will be described in the following section.

The rest of the text is organized as follow. First, we describe the characteristics of the mathematical models developed in this paper and the numerical methods employed in the implementation. Then, we present the results of computational simulations with the models and, finally, we discuss limitations and future work and draw our conclusions.

## 2. Materials and methods

This paper introduces a mathematical model composed of a system of Partial Differential Equations (PDEs) to describe the abscess formation. PDE-based models usually include terms such as growth, death and interaction terms and they have terms that are responsible for modeling the movement of cells, molecules and bacteria through the diffusion process. The majority of PDEs presented in this paper have the following structure in common:

(1) $$\begin{array}{l} \;\frac{\partial u}{\partial t}=fg+D\nabla \cdot \left(g\nabla u\right),\\ u\left(x,0\right)=u_{0},\frac{\partial u\left(.,t\right)}{\partial \vec{n}}{|}_{\partial \Omega }=0, \end{array}$$

where *u* is a variable that refers to a given population, the term *f* is a function that models the growth of *u* and the term *D*∇·(*g*∇*u*) models the nonlinear diffusion of *u*. Function *g* is equivalent to the *g* function proposed in (Painter and Sherratt, 2003). This function was originally developed to model the movement of interacting cell populations (Painter and Sherratt, 2003). We extended it to model interactions that also occur in other cellular processes. For example, we use the *g* function to model interactions that occur during bacterial growth or neutrophil migration. The *g* function is used to account for different interaction strengths between the populations and the effects of these in processes of growth, phagocytosis, migration, death and diffusion.

The *g* function is defined as the heaviside function of $\bar{g}$:

(2) $$\begin{array}{l} g\left(w\right)=\{\begin{array}{c} \bar{g}\left(w\right),\;0\leq \bar{g}\left(w\right)\leq 1\;\\ 0,\;otherwise.\; \end{array} \end{array}$$

Function $\bar{g}\left(w\right)$ is defined as:

(3) $$\begin{array}{l} \bar{g}\left(w\right)=1-\frac{w}{total}, \end{array}$$

where *w* is a term that models the interactions between distinct populations and *total* is a parameter that denotes the maximum population supported in a discretized region of the domain. In this work, we consider that the value of *total* is constant and is equal to 1 for all discretized regions.

The interactions between the populations can be stimulatory or inhibitory. In this paper, we consider only inhibitory interactions in the *w* term. To illustrate the meaning of *w*, consider, for example, a system with two types of populations: *u* and *v*. The interactions that each population has with the other one are modeled by the *w* term. Therefore, the *w* term is defined for each distinct population in the system. For example, the *w* for the *u* population is defined as:

(4) $$\begin{array}{l} w_{u}=w_{uu}u+w_{vu}v, \end{array}$$

where *w*_*uu*_*u* is the inhibition that *u* exerts on itself and *w*_*vu*_*v* is the inhibition that *v* exerts on *u*. These inhibitory relations will affect all processes in *u* dynamics. *w*_*uu*_ and *w*_*vu*_ are constant parameters. We call these parameters “weights” to refer to the fact that they control the strength of the inhibition that one population exerts on the other.

The $\bar{g}$ function for the *u* population is:

(5) $$\begin{array}{l} \bar{g}\left(w_{u}\right)=1-w_{u}. \end{array}$$

For the *v* population, we have:

(6) $$w_{v}=w_{vv}v+w_{u,v}u,$$

where *w*_*vv*_*v* is the inhibition that *v* exerts on itself and *w*_*uv*_*u* is the inhibition that *u* exerts on *v*. These inhibitory relations will affect all processes in *v* dynamics. *w*_*vv*_ and *w*_*uv*_ are constant parameters. The $\bar{g}$ function for the *v* population is:

(7) $$\begin{array}{l} \bar{g}\left(w_{v}\right)=1-w_{v}. \end{array}$$

We can extend the definition of *w* for a system with *n* distinct populations. Considering the *u* population again, *w*_*u*_ is defined as:

(8) $$w_{u}=w_{uu}u+\sum_{\begin{array}{l} j\in C,\\ j\neq u \end{array}}w_{ju}j,$$

where *C* is the set of all distinct populations in the system and *j* is one of these populations that is different from *u*. The summation accounts for the inhibition that *u* suffers from all other populations, with *w*_*ju*_ being the strength of the inhibition that *j* population exerts on *u*.

We can also interpret the *g* function as a way to model the effect that the lack of space has in the dynamics of a population because its value can be seen as the amount of available space in a discretized region of the domain. Considering that all regions in the domain support a maximum number of cells, molecules and/or bacteria (denoted by *total*), diffusion cannot occur for fully occupied regions where there is no available space. In these regions, we have *w* ≥ *total* which implies that $\frac{w}{total}\geq 1$ and $\bar{g}\left(w\right)\leq 0$ and, as a result, *g*(*w*) of Equation 2 is zero.

The diffusion of bacteria has another term, *h*(*b*), that models their cooperative behavior. The bacteria diffusion term is defined as:

(9) $$\begin{array}{l} D_{b}\nabla \cdot \left(g_{b}\left(w_{b}\right)h\left(b\right)\nabla b\right), \end{array}$$

where *g*_*b*_(*w*_*b*_) is the bacteria *g* function and *w*_*b*_ is the bacteria interaction term. The function *h*(*b*) models a behavior where the bacteria colony grows when conditions are favorable and the colony density is high. The bacteria will only colonize nearby regions when they were successful in establishing a colony in their current location. As a consequence of this, in our model, the diffusion of bacteria only occurs when bacteria concentration is above a threshold. The function *h*(*b*) is defined as:

(10) $$\begin{array}{l} h\left(b\right)=\frac{\left(\alpha +1\right)b^{\gamma }}{\alpha +b^{\gamma }}. \end{array}$$

This equation is a hyperbolic saturation function (Haefner, 2005) and it is known as Hill equation in this form (Goutelle et al., 2008). The Hill equation is used, for example, to model the relationship between drug concentration and its effects (Wagner, 1968). In this equation, the term α + 1 scales the maximum value to which the function is asymptotic, parameter α is a half saturation constant and γ is a shape parameter (Haefner, 2005). It is important to mention that the term *h*(*b*) is only present in the diffusion of bacteria. If we consider that the cooperative behavior is absent by doing *h*(*h*) = 1, we have a situation where, even for a region with very few bacteria, the bacteria can diffuse to neighboring regions with available space and, as a result, it is hard for the bacteria to form a colony surrounded by fibrin because some bacteria will always “escape.” Therefore, in our model, such cooperative behavior as well as the nonlinear diffusion are important to the formation of the abscess pattern.

In all models, the exchange between the vascular system (arterioles and vessels) and the tissue was assumed to occur in all points of the one-dimensional space. This is a reasonable first approach because the kidney is highly vascularized.

The numerical methods used were the following: (1) explicit Euler method for time discretization; and (2) for spatial discretization, we used the Finite Volume Method (FVM) (Versteeg and Malalasekera, 2011). The nonlinear diffusion was implemented with a method based on FVM, where the calculus of the divergent operator is based on the quantities calculated at the two interfaces (left and right) of the finite volume. The derivatives and the gradient operator are approximated with numerical fluxes calculated at the interfaces. The quantities at each interface are an average of the quantities on the neighboring nodes. In summary, FVM is based on the evaluation of influx and outflux in a control volume around each node in the mesh. The code was implemented in C and the graphs were generated with a script in Python.

## 3. Results

**Figure 2:**
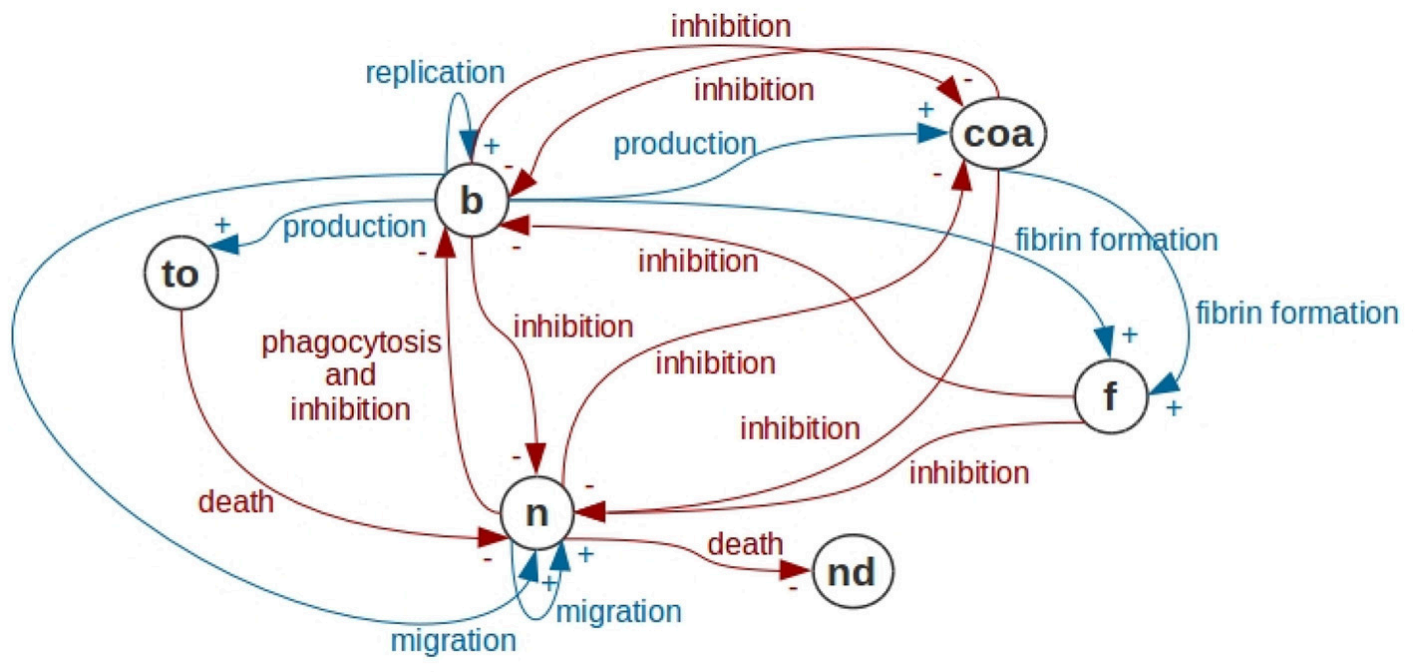
Interactions in the abscess formation model. In this figure, we use the notation of Causal Loop Diagrams (CLD) of System Dynamics. Bacteria are represented by *b*, Coa/vWbp are represented by *coa*, fibrin is represented by *f*, neutrophils are represented by *n*, dead neutrophils are represented by *nd* and toxins are represented by *to*. Bacteria have a replication process forming new bacteria. Bacteria produce Coa/vWbp and participate together with Coa/vWbp in fibrin network formation. Bacteria are phagocytosed by neutrophils. In addition, bacteria produce toxins that cause neutrophil death and inhibit all processes in neutrophil dynamics. The processes present in the bacteria dynamics are all inhibited by Coa/vWbp, fibrin and neutrophil. Coa production is inhibited by the neutrophil and by the bacteria. Neutrophil migration depends on neutrophils and on bacteria. All processes in neutrophil dynamics are inhibited by bacteria, fibrin and Coa/vWbp. In this diagram, we are not representing self-inhibitions that are also present in the mathematical models of this paper.

In this paper, we incrementally build a mathematical model of abscess formation. The interactions between the model's components are depicted in Figure 2. It is important to highlight that the intensity of a particular inhibitory relation (in Figure 2, inhibitory relations are represented by red arrows with the word inhibition) depends on concentrations of the cellular types that are exerting the inhibition. In the next sections, we will discuss each of these relations and we will present the characteristics of each submodel that is part of the abscess formation model.

### 3.1. Bacteria-Coa/vWbp-fibrin model

The first model accounts for the interaction between bacteria, Coa/vWbp and fibrin. The objective of this model is to reproduce the formation of a fibrin network around the bacteria colony. In this model, we have the bacteria replicating and producing two coagulation factors: *coagulase* (Coa) and von Willebrand factor Binding Protein (*vWbp*). These coagulation factors are responsible for converting fibrinogen into fibrin.

The model is comprised by the following system of equations:

(11) $$\begin{array}{ll} \frac{\partial coa}{\partial t} & =kbg_{coa}\left(b,f,coa\right)+D_{coa}\text{coa\_diffusion()},\\ coa\left(x,0\right) & =coa_{0},\frac{\partial coa\left(.,t\right)}{\partial \vec{n}}{|}_{\partial \Omega }=0,\\ \frac{\partial b}{\partial t} & =rbg_{b}\left(b,f,coa\right)+D_{b}\text{b\_diffusion()},\\ b\left(x,0\right) & =b_{0},\frac{\partial b\left(.,t\right)}{\partial \vec{n}}{|}_{\partial \Omega }=0,\\ f & =bcoa, \end{array}$$

where the term *coa* denotes the coagulation factors Coa and vWbp, *b* denotes the bacteria and *f* denotes fibrin. The functions *g*_*coa*_(*b, f, coa*) and *g*_*b*_(*b, f, coa*) are the *g* functions of Coa and bacteria, respectively. The functions coa_diffusion() and b_diffusion() models Coa/vWbp and bacteria diffusion, respectively. The diffusion is modeled in two ways: (1) with the classic diffusion operator (diffusion terms in the System of Equation 11); and (2) with the nonlinear diffusion given by 12 and 13. In the next section, we show the simulation results with both diffusion operators. Diffusion is the net movement of molecules or atoms from a region of high concentration (or high chemical potential) to a region of low concentration (or low chemical potential) as a result of random motion of the molecules or atoms.

The equation *f* = *bcoa* models fibrin formation. We assume that fibrin formation depends on the interaction between the bacteria and the coagulation factors.

The term *k*.*b*.*g*_*coa*_(*b, f, coa*) denotes the Coa/vWbp production, where *k* is the production rate. The function *g*_*coa*_(*b, f, coa*) is given by:

(12) $$\begin{array}{l} g_{coa}\left(b,f,coa\right)=1-\left(w_{bcoa}b+w_{fcoa}f+w_{coacoa}coa\right). \end{array}$$

The parameters *w*_*bcoa*_, *w*_*coacoa*_ and *w*_*fcoa*_ represent the influence of bacteria, Coa/vWbp and fibrin in Coa/vWbp dynamics.

The Coa/vWbp production is limited by the available space and is inhibited by bacteria and Coa/vWbp molecules that are in the same discretized region. This inhibition is considered to simulate the coagulation factors spreading from the border of the bacteria colony and also to simulate the fibrin network formation on this border.

The term *r*.*b*.*g*_*b*_(*b, f, coa*) denotes the bacteria replication, where *r* is the replication rate. The function *g*_*b*_(*b, f, coa*) is given by:

(13) $$\begin{array}{l} g_{b}\left(b,f,coa\right)=1-\left(w_{bb}b+w_{fb}f+w_{coab}coa\right). \end{array}$$

The parameters *w*_*bb*_, *w*_*coab*_ and *w*_*fb*_ represent the influence of bacteria, Coa/vWbp and fibrin in bacteria dynamics.

The bacteria replication is limited by the available space and is inhibited by Coa/vWbp molecules and the fibrin network. The Coa/vWbp inhibition is justified by the fact that, when the colony is being formed, the bacteria inside the colony will alter their behavior and, consequently, will decrease replication and focus on protecting themselves with the fibrin network. The fibrin network inhibition is considered to simulate that bacteria colony cannot replicate and expand over fibrin to other regions after the formation of the fibrin network.

#### 3.1.1. One-dimensional simulations

With the objective of understanding the spatiotemporal behavior of the bacteria-Coa/vWbp-fibrin model, the diffusion process was added to the model (Equation 11) and numerical simulations were carried out on a one-dimensional domain:

(14) $$\begin{array}{ll} \frac{\partial coa}{\partial t} & =kbg_{coa}\left(b,f,coa\right)+D_{coa}\frac{\partial^{2}coa}{\partial x^{2}}\\ coa\left(x,0\right) & =coa_{0},\frac{\partial coa\left(.,t\right)}{\partial \vec{n}}{|}_{\partial \Omega }=0\\ \frac{\partial b}{\partial t} & =rbg_{b}\left(b,f,coa\right)+D_{b}\frac{\partial^{2}b}{\partial x^{2}}\\ b\left(x,0\right) & =b_{0},\frac{\partial b\left(.,t\right)}{\partial \vec{n}}{|}_{\partial \Omega }=0\\ f & =bcoa \end{array}$$

$D_{coa}\frac{\partial^{2}coa}{\partial x^{2}}$ and $D_{b}\frac{\partial^{2}b}{\partial x^{2}}$ are the diffusion terms of Coa/vWbp and bacteria, respectively, where *D*_*coa*_ and *D*_*b*_ are the diffusion coefficients.

In spite of *S. aureus* not being a motile organism, we considered a diffusion process for *S. aureus* to simulate the bacterial expansion as the bacteria replicate and increase in number, having as a consequence an increase in the region occupied by the bacteria colony. We chose a small diffusion coefficient for the bacteria (*D*_*b*_ = 0.05) to simulate the aforementioned aspect of *S. aureus* infections.

The model's initial conditions and parameters are presented in Tables 1, 2, respectively. In our simulations, we assumed a one-dimensional domain of 10 *mm* length and a simulation time of 20 days. In fact, this one-dimensional model is a simplification of a 3D block model in that we have assumed that the lengths associated with *y* and *z* are much smaller than the length associated with *x*. In all PDEs, the domain is homogeneous and the boundary conditions are of Neumann type.

**Table 1:** Initial conditions.

**Variable**	**Value**	**Unit**
*b*_0_	$\{\begin{array}{l} 0.6:\;4\leq x\leq 6\\ 0:\;otherwise \end{array}$	amount/mm^3^
*n*_0_	0.01:0 ≤ *x* ≤ 10	amount/mm^3^
*coa*_0_	0.01:0 ≤ *x* ≤ 10	amount/mm^3^
*f*_0_	0:0 ≤ *x* ≤ 10	amount/mm^3^
*nd*_0_	0:0 ≤ *x* ≤ 10	amount/mm^3^
*to*_0_	0:0 ≤ *x* ≤ 10	amount/mm^3^

*The amount refers to the amount of one particular population (e.g., in b_0_ it refers to bacteria, in n_0_ it refers to neutrophils, and so on)*.

**Table 2:** Set of parameters used in simulations.

**Parameter**	**Value**	**Unit**
*r*	1.3	1/day
α	0.1	dimensionless
γ	5	dimensionless
*k*	2	1/day
*D*_*coa*_	0.05	mm/day
*s*	10	1/((amount/mm^3^).day)
*l*	40	1/((amount/mm^3^).day)
*D*_*n*_	3	mm/day
*w*_*bb*_	1	1/(amount/mm^3^)
*w*_*coab*_	4	1/(amount/mm^3^)
*w*_*nb*_	1.1	1/(amount/mm^3^)
*w*_*fb*_	1	1/(amount/mm^3^)
*w*_*bcoa*_	1.5	1/(amount/mm^3^)
*w*_*coacoa*_	1	1/(amount/mm^3^)
*w*_*ncoa*_	1.2	1/(amount/mm^3^)
*w*_*fcoa*_	0	1/(amount/mm^3^)
*w*_*bn*_	1.2	1/(amount/mm^3^)
*w*_*coan*_	0.5	1/(amount/mm^3^)
*w*_*nn*_	1	1/(amount/mm^3^)
*w*_*fn*_	2	1/(amount/mm^3^)
β_*to*_	0.5	1/((amount/mm^3^).day)
μ_*to*_	0.5	1/day
*D*_*to*_	2	mm/day
α_*to*_	0.7	1/((amount/mm^3^).day)

Bacteria are initially placed in the middle of the domain, neutrophils and the coagulation factors are placed initially with a small concentration all over the domain. The bacteria initial location can be seen as the set of points (arterioles) where bacteria extravasate from the vasculature to the kidney tissue.

In all computational simulations we used the parameters values presented in Table 2, except when we vary some parameters to simulate different scenarios and, in these cases, we highlight what are the new values employed.

Due to the lack of experimental data and the difficult in making a direct correlation between some measured biological quantities and the parameters of the models, the parameters values were chosen to illustrate the different behaviors that the models are capable of reproducing.

**Figure 3:**
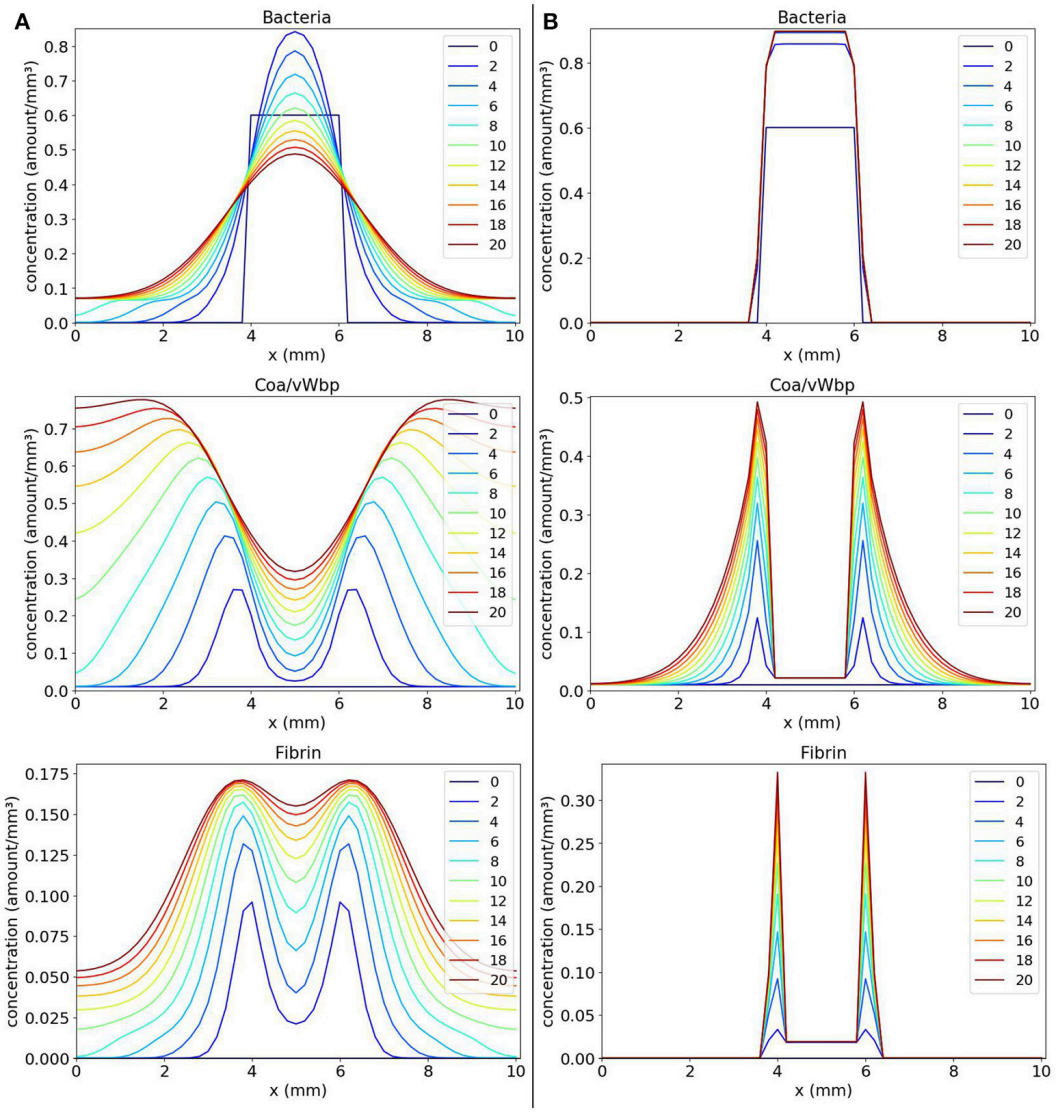
Spatial distribution of bacteria, Coa/vWbp and fibrin concentrations in the comparison between the classic (**A** on the left) and the nonlinear (**B** on the right) diffusion scenarios. The y-axis (concentration) represents the fraction occupied by a particular population in a discretized region of the domain. The x-axis (x) represents the space in *mm*. The simulated time correspond to 20 days. Each line represents a particular day. The simulation starts at day 0 and finishes at day 20. In classic diffusion scenario **(A)**, it is observed that the bacteria spread throughout the domain and cannot establish a colony surrounded by fibrin as in the nonlinear diffusion scenario **(B)**.

We observe in Figure 3A that, with time, the bacteria replicate and the bacteria colony increases in size. As a result, the production of the coagulation factors Coa/vWbp increases. With time, Coa/vWbp is converted to fibrin. The fibrin has some influence in bacteria's growth but fibrin was not able to prevent the spread of bacteria around the initial site of infection. We believe this happened because fibrin is not influencing bacteria diffusion as it influences bacterial growth. Therefore the bacteria colony can spread to other areas of the tissue. The spatial pattern seen in this result does not resemble the abscess pattern because we cannot observe the formation of one or more colonies of bacteria surrounded by fibrin.

In the simulated scenario described previously, we implemented the classical diffusion operator that does not consider any external influence in the diffusion of a population. In some situations, this hypothesis that the diffusion of a cell is not influenced by any other cell or molecule present in the system is not true. In the human body, a cell can interact with dozens of cells in a short period of time. Due to this fact, a cell can have many of its processes influenced by these interactions. Besides, space in the body is limited therefore the volume of some part of a tissue supports a maximum concentration of cells, molecules, liquids and other substances. The nonlinear diffusion models the influence of a cell population in the diffusion of other cell population. To represent the influence of both fibrin and Coa/vWbp in bacteria diffusion, the diffusion term of bacteria is defined as:

(15) $$\begin{array}{l} D_{b}\nabla \cdot \left(g_{b}\left(b,f,coa\right)h\left(b\right)\nabla b\right), \end{array}$$

where *g*_*b*_(*b, f, coa*) models the influence resulting from the interactions between bacteria, Coa/vWbp and fibrin. The term *h*(*b*) models the cooperative behavior of bacteria and was defined in Equation 7. The diffusion of Coa/vWbp is defined as:

(16) $$\begin{array}{l} D_{coa}\nabla \cdot \left(g_{coa}\left(b,f,coa\right)\nabla coa\right). \end{array}$$

The nonlinear diffusion simulates the fact that bacteria colonies will be unable to expand to some points where fibrin concentration is sufficiently high reproducing, in this way, the formation of a fibrin network around the colonies. The fibrin network acts like a barrier preventing any cell to cross it. We will show that these hypotheses are important in the development of a mathematical model of abscess formation.

Incorporating the nonlinear diffusion terms in the PDEs, we obtain the following system:

(17) $$\begin{array}{ll} \frac{\partial coa}{\partial t} & =kbg_{coa}\left(b,f,coa\right)+D_{coa}\nabla \cdot \left(g_{coa}\left(b,f,coa\right)\nabla coa\right),\\ \frac{\partial b}{\partial t} & =rbg_{b}\left(b,f,coa\right)+D_{b}\nabla \cdot \left(g_{b}\left(b,f,coa\right)h\left(b\right)\nabla b\right),\\ f & =bcoa. \end{array}$$

The results obtained with numerical simulations of these equations are shown in Figure 3B. We observe that, initially, the bacteria colony grows and starts to expand. At the same time, the bacteria produce the coagulation factors Coa/vWbp. The concentration of these factors increases and they convert fibrinogen, present in the body and that is not explicitly considered here, to fibrin. In addition, the fibrin concentration increases and we can see that fibrin is located around the bacteria colony. Both coagulation factors and fibrin interacts with bacteria preventing them to colonize other parts of the tissue. This process reflects the quorum sensing behavior seen in *S. aureus* infections.

Quorum sensing (Painter and Hillen, 2002; Yarwood and Schlievert, 2003; Le and Otto, 2015) is the process by which microorganisms regulate population density through chemical signaling. Chemical molecules secreted by microorganisms are a form of intra- and interspecies communication that helps bacteria coordinate their behavior. Quorum sensing allows to modulate diverse characteristics of the microorganisms, such as the motility, production of virulence factors and the formation of biofilms. In staphylococci, the ability to sense the bacterial density, or quorum, and to respond with genetic adaptations is an important mechanism to bacteria survival in the host (Le and Otto, 2015).

The nonlinear diffusion improved the model result, making it possible to obtain a pattern more similar to an abscess. However, abscesses are also composed by dead and live neutrophils. To reproduce the complete pattern, it is necessary to include these types of cell in the model. We will start including live neutrophils, and then dead neutrophils and toxins will be included. We will use the PDEs system given by Equations 14 as a base for further developments of our mathematical model of abscess formation.

### 3.2. Bacteria-neutrophil model

The model of interaction between bacteria and neutrophil, called bacteria-neutrophil model, is similar to the bacteria-Coa/vWbp-fibrin model presented previously in section 3.1. The neutrophil migration depends on bacteria concentration as the production of Coa/vWbp. The neutrophil has also a *g* function that is present in both growth and diffusion terms.

The bacteria-neutrophil model is comprised by the following set of PDEs:

(18) $$\begin{array}{l} \frac{\partial b}{\partial t}=(r-ln)bg_{b}(b,n)+D_{b}\nabla \cdot (g_{b}(b,n)h(b)\nabla b)),\\ \frac{\partial n}{\partial t}=sbng_{n}(b,n)+D_{n}\nabla \cdot (g_{n}(b,n)\nabla n)). \end{array}$$

The variable *n* denotes neutrophil concentration and the variable *b* denotes bacteria concentration. The term *s*.*b*.*n*.*g*_*n*_(*b, n*) models neutrophil migration. Product *b*.*n* in term *s*.*b*.*n*.*g*_*n*_(*b, n*) can be interpreted as the pro-inflammatory cytokine production. The pro-inflammatory cytokines would have the effect of attracting more neutrophils to the infection site. For the sake of simplicity, these cytokines are not considered explicitly in this model. The term *r*.*b*.*g*_*b*_(*b, n*) represents bacteria replication. Bacteria phagocytosis is denoted by the term *l*.*n*.*b*.*g*_*b*_(*b, n*). The model has two *g* functions: (1) *g*_*b*_(*b, n*) for bacteria; and (2) *g*_*n*_(*b, n*) for neutrophils.

The *g* functions equations are given by:

(19) $$\begin{array}{l} g_{b}\left(b,n\right)=1-\left(w_{bb}.b+w_{nb}.n\right),\\ g_{n}\left(b,n\right)=1-\left(w_{bn}.b+w_{nn}.n\right). \end{array}$$

The model's parameters are: (1) *r* is the bacteria replication rate; (2) *l* is the phagocytosis rate; (3) *w*_*bb*_ is the influence of bacteria on its own dynamics; (4) *w*_*nb*_ is the influence of neutrophils on bacteria dynamics; (5) *s* is the neutrophil migration rate; (6) *w*_*bn*_ is the influence of bacteria on neutrophils dynamics; and (7) *w*_*nn*_ is the influence of neutrophils on its own dynamics.

#### 3.2.1. One-dimensional simulations

With the objective of analyzing the spatiotemporal behavior of bacteria-neutrophil model, one-dimensional simulations of the Equation 15 were performed. In the simulations performed, we observed three main behaviors: (1) the formation of a bacteria colony when considering a small phagocytosis rate; (2) a disseminated infection when small rates for phagocytosis and for neutrophil migration are considered; and (3) infection control with complete elimination of bacteria when considering a “normal” immune response.

**Figure 4:**
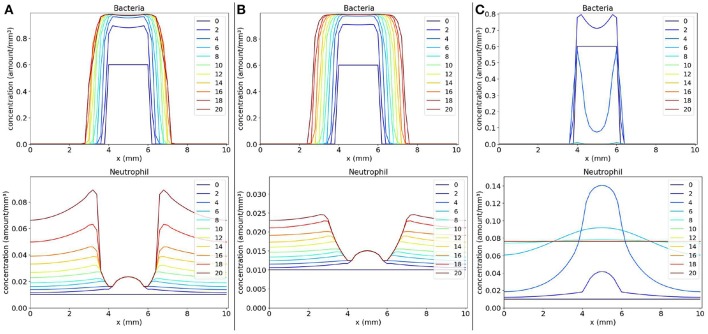
Spatial distribution of bacteria and neutrophil concentrations in three distinct scenarios: (a) the scenario with small phagocytosis rate (**A** on the left), (b) the scenario with small rates for phagocytosis and neutrophil migration (**B** on the middle), and (c) the scenario with “normal” values for phagocytosis and neutrophil migration (**C** on the right). The y-axis (concentration) represents the fraction occupied by a particular population in a discretized region of the domain. The x-axis (x) represents the space in *mm*. The simulated time correspond to 20 days. Each line represents a particular day. The simulation starts at day 0 and finishes at day 20. In **(A)**, we observe that the bacteria colony grows and infects other regions because the neutrophil response is very ineffective. The same occurs in **(B)** where, besides an impairment in phagocytosis, there are very few neutrophils to fight the infection. A different situation occurs in **(C)** where neutrophils are capable of eliminating bacteria completely, controlling the infection.

Values of parameters *s* (rate of neutrophil migration) and *l* (rate of phagocytosis) were varied in three different scenarios: (1) small phagocytosis rate: *s* = 10 and *l* = 20 (Figure 4A); (2) small rates for phagocytosis and neutrophil migration: *s* = 5 and *l* = 20 (Figure 4B); and (3) “normal” values for phagocytosis and neutrophil migration: *s* = 10 and *l* = 40 (Figure 4C).

The first scenario is presented in Figure 4A. This scenario simulates the mechanisms employed by bacteria to escape phagocytosis by immune cells. We observe that neutrophils begin to migrate to the tissue in an attempt to control the infection, but they are not able to phagocytose bacteria efficiently. As a consequence, the bacteria colony grows and expands around the initial site of infection.

The second scenario (Figure 4B) simulates a deficient immune response where it is considered an impairment in neutrophil migration caused by bacteria, besides the impairment in phagocytosis. It is observed that the bacteria colony can rapidly expand to other areas of the tissue without the presence of neutrophils. Neutrophil migration is impaired and there are almost no neutrophils to fight the infection. Eventually, with time, the bacteria will spread to larger areas of the tissue.

In the last simulated scenario (Figure 4C), we considered a normal immune response. We observe that the bacteria were completely eliminated by neutrophils. Neutrophils were successful in controlling the infection due to rapid migration and efficient killing of bacteria. After bacteria elimination, the spatial distribution of neutrophils tend to stabilize throughout the tissue due to the fact that we have not modeled the neutrophil apoptosis.

### 3.3. Bacteria-Coa/vWbp-fibrin-neutrophil model

The bacteria-Coa/vWbp-fibrin-neutrophil model is an extension combining the two models presented previously: the bacteria-Coa/vWbp-fibrin model and the bacteria-neutrophil model. The objective of this model is to reproduce, in addition to the formation of one or more colonies of bacteria surrounded by fibrin, the spatial distribution of neutrophils inside the abscess lesion. The model is comprised by the following PDEs system:

(20) $$\begin{array}{l} \frac{\partial coa}{\partial t}=kbg_{coa}\left(b,f,coa,n\right)+D_{coa}\nabla \cdot \left(g_{coa}\left(b,f,coa,n\right)\nabla coa\right),\\ \frac{\partial b}{\partial t}=\left(r-ln\right)bg_{b}\left(b,f,coa,n\right)+D_{b}\nabla \cdot \left(g_{b}\left(b,f,coa,n\right)h\left(b\right)\nabla b\right),\\ f=bcoa,\\ \frac{\partial n}{\partial t}=sbng_{n}\left(b,f,coa,n\right)+D_{n}\nabla \cdot \left(g_{n}\left(b,f,coa,n\right)\nabla n\right). \end{array}$$

The equation *f* = *b coa* models fibrin formation. The *g* functions now depend on four types of populations: bacteria, Coa/vWbp, fibrin and neutrophil. The new *g* functions are given by:

(21) $$\begin{array}{l} g_{coa}\left(b,f,coa,n\right)=\left(1-w_{bcoa}b-w_{fcoa}f-w_{coacoa}coa-w_{ncoa}n\right),\\ g_{b}\left(b,f,coa,n\right)=\left(1-w_{bb}b-w_{fb}f-w_{coab}coa-w_{nb}n\right),\\ g_{n}\left(b,f,coa,n\right)=\left(1-w_{bn}b-w_{fn}f-w_{coan}coa-w_{nn}n\right). \end{array}$$

It is important to highlight that when choosing *n* = 0 in Equation 17, we obtain the bacteria-Coa/vWbp-fibrin model presented in Equation 14. In addition, when we consider *coa* = 0 and *f* = 0 in Equation 17, we obtain the bacteria-neutrophil model presented in Equation 15.

The parameters of the model are: (1) *k* is the Coa/vWbp production rate; (2) *r* is the rate of bacteria replication; (3) *l* is the rate of phagocytosis; (4) *s* is the neutrophil migration rate; (5) *w*_*bcoa*_, *w*_*fcoa*_, *w*_*coacoa*_ and *w*_*ncoa*_ are the influence of bacteria, fibrin, Coa/vWbp and neutrophil, respectively, in Coa/vWbp dynamics; (6) *w*_*bb*_, *w*_*fb*_, *w*_*coab*_ and *w*_*nb*_ are the influence of bacteria, fibrin, Coa/vWbp and neutrophil, respectively, in bacteria dynamics; (7) *w*_*bn*_, *w*_*fn*_, *w*_*coan*_ and *w*_*nn*_ are the influence of bacteria, fibrin, Coa/vWbp and neutrophil, respectively, in neutrophil dynamics; and (8) *D*_*coa*_, *D*_*b*_ and *D*_*n*_ are the diffusion coefficients of Coa/vWbp, bacteria and neutrophil, respectively.

In this model, we consider, besides fibrin influence in the dynamics of bacteria, also their influence in the dynamics of neutrophil. The influence is reflected in the fact that when fibrin concentration is sufficiently high, fibrin prevents neutrophils from getting closer to the bacteria colonies. It is important to highlight that phagocytosis is also influenced by fibrin. Depending on fibrin's location in the domain, for example, if fibrin is located around a bacteria colony it will protect bacteria from being phagocytized by neutrophils outside the colony. Neutrophils inside the colony are not capable of handling the infection alone.

#### 3.3.1. One-dimensional simulations

We first present and compare the results of two scenarios: (1) a scenario with the coagulation factors production rate *k* equals to 2; and (2) a scenario with the coagulation factors production rate *k* equals to 0.4.

**Figure 5:**
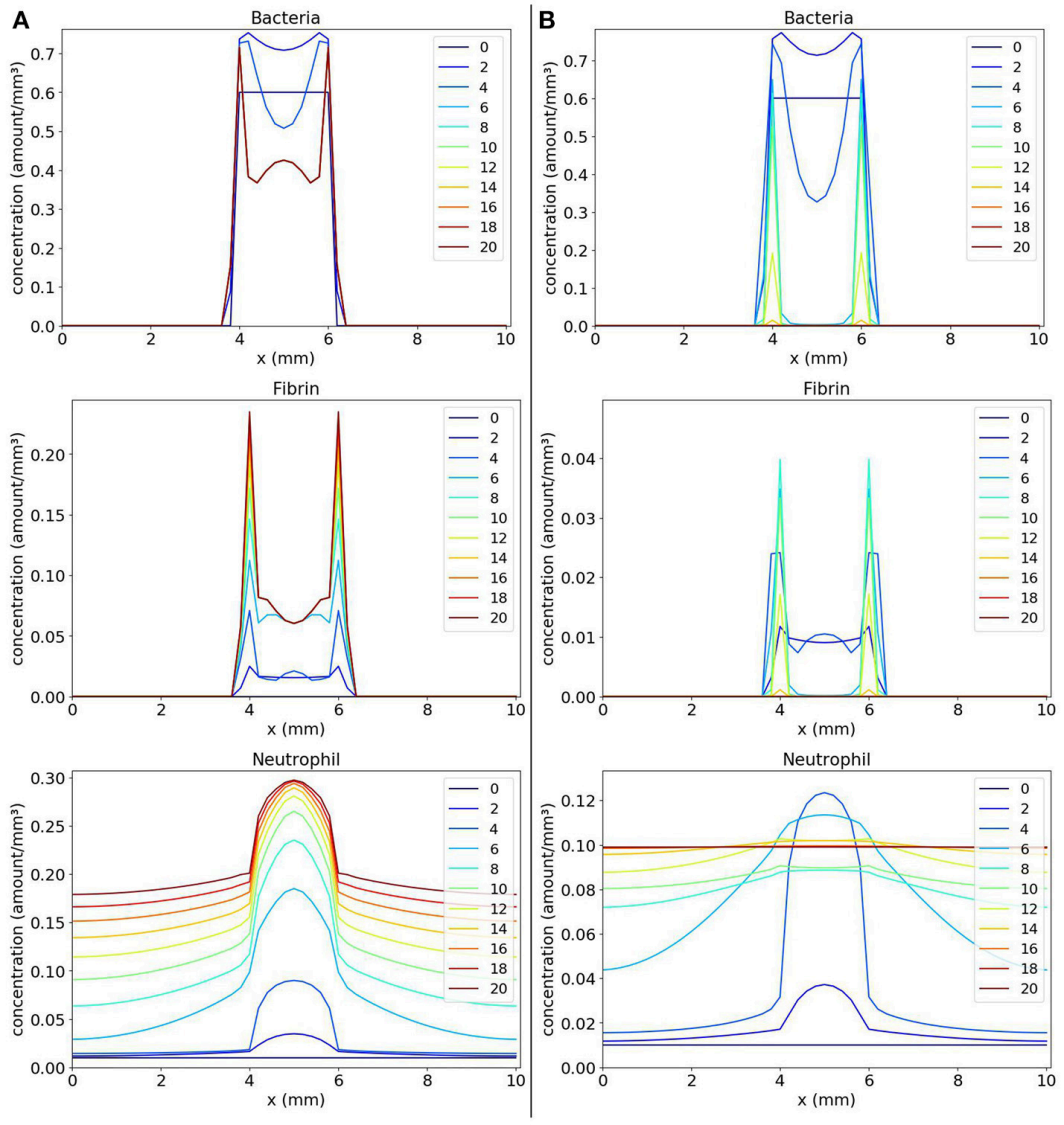
Spatial distribution of bacteria, fibrin and neutrophil concentrations in the comparison between the scenario with *k* = 2 (**A** on the left) and the scenario with *k* = 0.4 (**B** on the right). The y-axis (concentration) represents the fraction occupied by a particular population in a discretized region of the domain. The x-axis (x) represents the space in *mm*. The simulated time correspond to 20 days. Each line represents a particular day. The simulation starts at day 0 and finishes at day 20. **(A)** shows that the neutrophils that migrate into the tissue phagocytose part of the colony of bacteria until saturation occurs in regions where there are neutrophils and bacteria. At this time, no more phagocytosis occurs. In **(B)**, the colony of bacteria cannot produce fibrin fast enough to protect itself and it is eliminated.

The first scenario is presented in Figure 5A. We can observe that neutrophils have been able to enter the site of the colony of bacteria, but were not able to eliminate them after saturation of several points of the domain. The saturation ocurred also due to the production of the coagulation factors and fibrin formation. This scenario illustrates a limitation of the model: after saturation of a domain position, neutrophils cannot phagocytose bacteria there anymore. We observed that saturation occurred because parameter *w*_*bn*_ has a great impact in the model results together with the initial condition. If the product *w*_*bn*_.*b* is sufficiently high, in some points of the domain, few neutrophils can migrate to the tissue before it saturates. As a consequence, these neutrophils are not in sufficient number to eliminate all bacteria there. Another limitation is the fact that we are not considering any mechanism used by the bacteria to kill neutrophils. As a result, we have the stabilization of cells populations with a considerable amount of neutrophils inside the bacteria colony. These limitations were the primary motivation for the development of an extension of the current model by adding a variable that represents the toxins produced by the bacteria. Toxins are also important for the persistence of bacteria in the host. Basically, we can assume that toxins interact with neutrophils causing their death.

The second scenario (Figure 5B) shows that when we decrease the value of Coa/vWbp production (parameter *k*) to 0.4 and, consequently, decreasing the fibrin formation, the bacteria are completely eliminated. This scenario illustrates the importance of fibrin in protecting the bacteria.

The simulations with bacteria-Coa/vWbp-fibrin-neutrophil model allowed us to better understand the effect of each parameter in the dynamics of the model. We have observed that, for the immune response to be effective, the rate of neutrophil migration cannot be so high because the regions with bacteria could saturate rapidly and, in this case, neutrophils could no longer eliminate the bacteria. We have also observed that the rate of phagocytosis has an important role in model dynamics. The elimination of bacteria was only obtained when we considered a high phagocytosis rate combined with a moderate migration rate and a small rate for Coa/vWbp production.

### 3.4. Bacteria-Coa/vWbp-fibrin-neutrophil-toxin model

The previous model (Equation 17) can be modified to better understand the effects of toxins produced by *Staphylococcus aureus*. The toxins also contribute to the persistence of *S. aureus* in the host (Cheng et al., 2009, 2010). The role of toxins is to mantain cells of the immune system, mainly neutrophils, away from the colony of *S. aureus*. Even after the formation of the fibrin network, *S. aureus* bacteria continue to produce several types of toxins, which, because of their small volume, are able to pass through the fibrin network and reach the regions where most living neutrophils are migrating to the infected tissue.

It is important to highlight that the immune system of wild type mice as well as the immune system of humans is efficient in eliminating dead cells from tissue, cleaning the infection site. This cleansing would allow neutrophils and other immune system cells to approach the fibrin network around the bacterial colony, threatening to dissolve (to break down) this network to gain access to the colony of bacteria, but the toxins may prevent this process (Guggenberger et al., 2012; McAdow et al., 2012).

It was considered a simplified model of toxin's dynamics based on the following hypothesis:

- The production of toxins depends on bacteria concentration, having a saturation. This production is not influenced by other cells;
- The toxins cause the death of neutrophils at a rate that is proportional to the concentration of both;
- It is considered that the diffusion of toxins is not influenced by the presence of other cells;
- Both toxins and dead neutrophils do not influence the growth and diffusion of other cell types.

It is assumed that the volume of toxins and of dead neutrophils are negligible in relation to the volumes of other cells, therefore they are not considered in the *g* functions.

The model is composed by the following PDEs system:

(22) $$\begin{array}{l} \frac{\partial coa}{\partial t}=k.b.g_{coa}\left(b,f,coa,n\right)+D_{coa}\nabla \cdot \left(g_{coa}\left(b,f,coa,n\right).\nabla coa\right),\\ \frac{\partial b}{\partial t}=\left(r-l.n\right).b.g_{b}\left(b,f,coa,n\right)+D_{b}\nabla \cdot \left(g_{b}\left(b,f,coa,n\right).h\left(b\right).\nabla b\right),\\ f=bcoa,\\ \frac{\partial n}{\partial t}=s.b.n.g_{n}\left(b,f,coa,n\right)-\alpha_{to}.to.n+D_{n}\nabla \cdot \left(g_{n}\left(b,f,coa,n\right).\nabla n\right),\\ \frac{\partial to}{\partial t}=\beta_{to}.b.\left(1-to\right)-\mu_{to}.to+D_{to}.\Delta to,\\ \frac{\partial nd}{\partial t}=\alpha_{to}.to.n, \end{array}$$

where toxins represented by *to* and dead neutrophils represented by *nd* are the new populations added to the model. Term β_*to*_.*b*.(1−*to*) denotes toxin production, where β_*to*_ is the production rate. Term μ_*to*_.*to* denotes toxin decay and term *D*_*to*_.Δ*to* denotes toxin diffusion with μ_*to*_ being the decay rate and *D*_*to*_ being the diffusion coefficient. Neutrophils in contact with toxins die at a rate α_*to*_ that is proportional to the concentration of both (term α_*to*_.*to*.*n*). The *g* functions are the same as in the previous model.

#### 3.4.1. One-dimensional simulations

Simulations in one dimension were carried out to understand the new behaviors that can be obtained after the introduction of the toxin. In simulations with the toxin model, we have used the parameter values of the “normal” immune response (*s* = 10 and *l* = 40) scenario (Table 2) with the exception of Coa/vWbp production rate *k* which we varied in the two scenarios presented here. The values of the new parameters that were incorporated into the model are: β_*to*_ = 0.5, μ_*to*_ = 0.5, *D*_*to*_ = 2, and α_*to*_ = 0.7.

**Figure 6:**
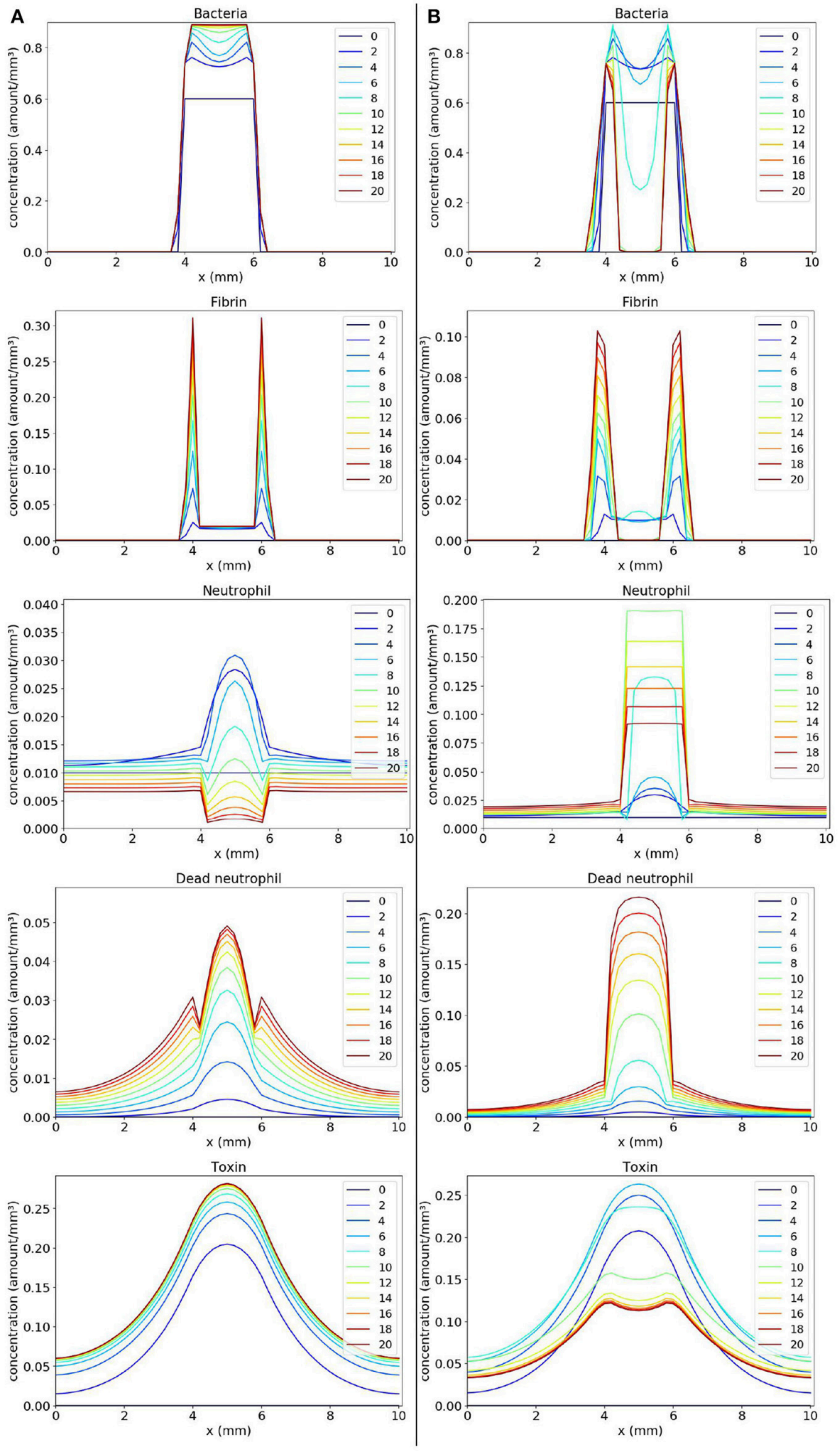
Spatial distribution of bacteria, fibrin, live neutrophil, dead neutrophil and toxin concentrations in the comparison between two scenarios: a scenario where the bacteria persist forming one colony **(A)** and a scenario where the bacteria persist forming two colonies **(B)**. The y-axis (concentration) represents the fraction occupied by a particular population in a discretized region of the domain. The x-axis (x) represents the space in *mm*. The simulated time correspond to 20 days. Each line represents a particular day. The simulation starts at day 0 and finishes at day 20. **(A)** shows that bacteria rapidly produce toxins killing neutrophils, and ensuring that they form a colony protected by fibrin. **(B)** shows a similar behavior, but, this time, neutrophils manage to phagocytose a great number of bacteria located near the middle of the domain. This results in a separation of the initial colony in two. These newly formed colonies have time to produce sufficient fibrin to protect themselves, surviving in the host.

In the first scenario presented in Figure 6A, we considered *k* = 2. We observe, in Panel A, that as the toxin diffuses through the tissue, it causes a lot of death in the region occupied by the bacteria colony. As a consequence, a concentration of dead neutrophils is observed at the infection site. The toxins helped bacteria to establish a favorable environment to persist.

One interesting result is observed when we consider a smaller Coa/vWbp production rate (*k* = 0.5) in second scenario (Figure 6B). In this case, we see the formation of two abscesses next to each other. Neutrophils migrate in the middle of the domain where the concentration of bacteria is high and phagocyte bacteria there. Neutrophils start to die due to the action of toxins. The toxins together with saturation after fibrin formation prevent neutrophils to eliminate bacteria completely and, as a result, there are the formation of two abscesses. In histology images of mice kidneys infected with *S. aureus*, it is also observed, in many situations, the formation of one or more abscesses (Cheng et al., 2009, 2010; Kim et al., 2011, 2012)

## 4. Discussion

In the mathematical models developed in this paper, we have considered the influence of a population on the dynamics of other population. This influence represents not only the lack of available space due to the volume occupied by distinct populations in a discretized region but also represents other types of interactions such as inhibitory or stimulatory interactions. These interactions are modeled through the use of the *g* function presented first in section 2. The interactions between different populations were modeled through the product of their concentration by constant parameters. We can also model these interactions by considering some function of various parameters. However, in order to avoid introducing complexity into the model and trying to better understand its behavior, we have chosen more simplified interactions.

Numerical simulations were important for us to understand the effects of the *g* function not only on the growth terms but also on the processes of movement. As shown in Figure 3A, without considering the *g* function in the diffusion terms, it was not possible to obtain a pattern similar to an abscess. This happened because bacteria and fibrin could move freely through the domain. There was nothing to stop them from moving to a location already containing a large concentration of cells, molecules and other substances. The incorporation of the *g* function into the diffusion, in this case, allowed us to model a behavior that is believed to be more real in this situation: an adaptive behavior in which populations adapt to the environment around them. This adaptation occurs due to the lack of space, but it could be due to the lack of nutrients, for example. With the *g* function, it is possible to react to changes in the environment avoiding a situation where more populations are created in a place where this creation would not be possible anymore.

We think that the effect of the *g* function on the movement of populations contributes to stabilize their spatial distributions. Studies on parasitoid–host interaction and on predator-prey models (Briggs and Hoopes, 2004, and references therein) found some spatial mechanisms resulting on stability or increased persistence. One of these mechanisms is the limited dispersion of populations. One of the effects of the *g* function, in this paper, is to limit the diffusion at the cellular level. In the case of models that consider patch dynamics, other important mechanisms that contribute to persistence are: spatial heterogeneity and asynchronous dynamics between patches (Briggs and Hoopes, 2004, and references therein).

It was possible to observe, with the simulations, that the parameter *w*_*bn*_ is important for the persistence of bacteria in the host, because this parameter represents the influence that the bacteria exert in neutrophil migration. The higher the value of *w*_*bn*_, the lower the migration of neutrophils and the lower the efficiency of neutrophil response. Another important parameter is the rate of neutrophil migration *s*. We have observed that this rate cannot be very high because a great concentration of neutrophils would saturate rapidly the regions with bacteria before eliminating them. But this rate cannot be small because bacteria would spread throughout the domain. The model results are also affected considerably by the rate of Coa/vWbp production *k*. If this rate is below a threshold then we have a scenario where bacteria are completely eliminated. Otherwise, we have a scenario where bacteria persist in the host.

### 4.1. Limitations and future work

As limitations of this paper, we can note the fact that the use of models based on differential equations requires detailed knowledge about the parameters that are included in the equations. Some of these parameters can be measured experimentally, while others need to be estimated. In this paper, we used parameter values for illustration purposes, they were not estimated due to lack of sufficient experimental data.

As future work, we plan to better study the effects of toxins and the different behaviors that could be obtained by considering it. We also plan to study the effect of considering the migration of cells ocurring only at some points of the domain, simulating the presence of blood vessels at those points. Some numerical simulations already performed using this specific scenario have shown that the chemotaxis process of neutrophils has a major impact in the result because the chemotaxis allows neutrophils to reach the bacterial colony faster than when diffusion only applies. This observation is in good agreement with our previous observations (Pigozzo et al., 2013). Besides, we plan to add pro-inflammatory cytokines to the model and to consider their chemoattractant effect on immune system cells.

As a future work, we plan to build a more complete model and validate it with distinct experimental data such as histology images, values of bacteria load in the tissue, size of abscess diameter, among others, obtained from various *in vivo* experiments including the leukocyte depletion experiment (Robertson et al., 2008; Navarini et al., 2009; Attia et al., 2013) and the Coa/vWbp inhibition experiment (Vanassche et al., 2011, 2012; Flick et al., 2013). We plan to consider, in our model, the use by the bacteria *S. aureus* of its sensory/regulatory systems to adapt the production of virulence factors, specifically to a triggering signal, e.g., neutrophils (Guerra et al., 2017). The idea is to study how the interaction between *S. aureus* and neutrophils provokes certain sensing and adaptive responses used by *S. aureus* (Guerra et al., 2017).

In addition, we plan to extend the model to two and three-dimensional domains, but we think that the behaviors that could be obtained with two or three dimensions are the same that we can obtain with the one-dimensional models because the spatial mechanisms considered are not altered with the increase in the number of dimensions.

## 5. Conclusions

In this paper, we have developed computational models based on partial differential equations that were able to reproduce some characteristics observed in the abscess formation process.

The study comprised the analysis of the spatiotemporal behavior of bacteria, the coagulation factors Coa/vWbp, fibrin, toxins and neutrophils. These analyses were important and helped to understand how the modeled processes interact, the effects of the incorporation of certain processes, among other factors.

It was shown, in this paper, that the use of the *g* function in the growth and diffusion terms of the populations was one of the characteristics that allowed the mathematical models to reproduce some key aspects of the abscess formation process. Other important characteristic was the fibrin network formation. The fibrin network protected bacteria from the immune response given by the neutrophils. The formation of the fibrin network was modeled considering the production of coagulation factors and the interaction of these factors with the colony of bacteria.

More tests and refinement of the model may be needed, but this initial model was capable of reproducing some characteristics found in the abscess pattern such as: the formation of a fibrin network around the colonies of bacteria and an accumulation of necrotic neutrophils and live neutrophils in the abscess region.

Based on simulations results and on analyses done so far, we believe that the fibrin network is essential for bacteria persistence inside the abscess lesion together with the mechanisms used by the bacteria to kill neutrophils such as the production of toxins and mechanisms used to evade phagocytosis.

The abscess pattern can also be obtained by models other than those based on PDEs. For example, Cellular Automata (Zorzenon dos Santos and Coutinho, 2001; Moreira and Deutsch, 2002; Xiao et al., 2006), Colored Petri Nets (Carvalho et al., 2015; Pennisi et al., 2016) and models based on Agents (Gopalakrishnan et al., 2013; Chiacchio et al., 2014; Abar et al., 2017) can also be used to capture this pattern of formation.

## Author contributions

DM have helped the understanding of histopathology of abscesses. AP, RS, ML, and SM have defined the methods and experiments. AP has written the software code to implement the model and has performed all simulations. AP, RS and ML have analyzed and interpreted the results. All authors have written, read and approved the final version of the paper.

### Conflict of interest statement

The authors declare that the research was conducted in the absence of any commercial or financial relationships that could be construed as a potential conflict of interest.

## Footnotes

^1^“Lysing toxins” or membrane-active toxins that interact with membranes of host cells and - under some conditions - can cause lysis of those cells.
